# Targeted Screening Strategies to Detect *Trypanosoma cruzi* Infection in Children

**DOI:** 10.1371/journal.pntd.0000103

**Published:** 2007-12-26

**Authors:** Michael Z. Levy, Vivian Kawai, Natalie M. Bowman, Lance A. Waller, Lilia Cabrera, Viviana V. Pinedo-Cancino, Amy E. Seitz, Frank J. Steurer, Juan G. Cornejo del Carpio, Eleazar Cordova-Benzaquen, James H. Maguire, Robert H. Gilman, Caryn Bern

**Affiliations:** 1 Division of Parasitic Diseases, Centers for Disease Control and Prevention, Atlanta, Georgia, United States of America; 2 Program in Population Biology, Ecology, and Evolution, Division of Biological and Biomedical Sciences, Emory University, Atlanta, Georgia, United States of America; 3 Asociación Benéfica Proyectos en Informática, Salud, Medicina y Agricultura, Lima, Perú; 4 Dirección Regional del Ministerio de Salud, Arequipa, Perú; 5 Department of Epidemiology and Preventive Medicine, University of Maryland, Baltimore, Maryland, United States of America; 6 Department of International Health, Bloomberg School of Public Health, Johns Hopkins University, Baltimore, Maryland, United States of America; Universidad de Buenos Aires, Argentina

## Abstract

**Background:**

Millions of people are infected with *Trypanosoma cruzi*, the causative agent of Chagas disease in Latin America. Anti-trypanosomal drug therapy can cure infected individuals, but treatment efficacy is highest early in infection. Vector control campaigns disrupt transmission of *T. cruzi*, but without timely diagnosis, children infected prior to vector control often miss the window of opportunity for effective chemotherapy.

**Methods and Findings:**

We performed a serological survey in children 2–18 years old living in a peri-urban community of Arequipa, Peru, and linked the results to entomologic, spatial and census data gathered during a vector control campaign. 23 of 433 (5.3% [95% CI 3.4–7.9]) children were confirmed seropositive for *T. cruzi* infection by two methods. Spatial analysis revealed that households with infected children were very tightly clustered within looser clusters of households with parasite-infected vectors. Bayesian hierarchical mixed models, which controlled for clustering of infection, showed that a child's risk of being seropositive increased by 20% per year of age and 4% per vector captured within the child's house. Receiver operator characteristic (ROC) plots of best-fit models suggest that more than 83% of infected children could be identified while testing only 22% of eligible children.

**Conclusions:**

We found evidence of spatially-focal vector-borne *T. cruzi* transmission in peri-urban Arequipa. Ongoing vector control campaigns, in addition to preventing further parasite transmission, facilitate the collection of data essential to identifying children at high risk of *T. cruzi* infection. Targeted screening strategies could make integration of diagnosis and treatment of children into Chagas disease control programs feasible in lower-resource settings.

## Author Summary

Chagas disease kills more people than any other parasitic disease in the Americas. The disease is caused by *Trypanosoma cruzi*, a single-cell parasite which is transmitted between people and other mammals by triatomine insects. Chagas disease control programs in Latin America have focused mainly on stopping transmission of *T. cruzi* rather than active surveillance for infection among human populations at risk. These programs have been very successful in controlling triatomine insects by spraying households with insecticides. This article is the first to describe *T.* cruzi transmission in an urban environment and show evidence that transmission may be epidemic in the city of Arequipa, Peru. The article also demonstrates how data easily collected during an ongoing insecticide spraying campaign in Arequipa might be used to identify children at greatest risk of infection with *T. cruzi*. The results of the analysis might aid in the optimal use of limited diagnostic resources by targeting screening efforts to those children in greatest need of diagnosis. Such targeted screening could facilitate the integration of diagnosis and treatment of children into Chagas disease control programs currently focused primarily on insects.

## Introduction

An estimated 11 million people are currently infected with the causative agent of Chagas disease, *Trypanosoma cruzi*, in Latin America [1],[2]. *T. cruzi* is a protozoan parasite carried in the gut of bloodsucking triatomine bugs (Hemiptera, Reduviidae), and humans become infected with the trypanosome mainly through contamination with the insect's feces deposited on mucous membranes or broken skin. Many countries have implemented Chagas disease control activities, though most focus on interruption of *T. cruzi* transmission rather than surveillance for infection among human populations at risk. *Triatoma infestans* is the principal vector of *T. cruzi* in South America and the sole vector in southern Peru. A campaign to eliminate *T. infestans*, known as the Southern Cone Initiative, has been remarkably successful in interrupting vector-borne transmission of *T. cruzi* through household insecticide application, especially in Uruguay, Chile and Brazil [1]. Although the World Health Organization recommends serologic diagnosis and drug treatment of all *T. cruzi*-infected children in affected areas, national control programs in Peru and other countries have not had sufficient resources for comprehensive serological screening [3].

Anti-trypanosomal drug therapy can cure 60% or more of infected children aged < 13 years [4],[5],[6], however treatment efficacy apparently decreases with the duration of infection and side effects to anti-trypanosomal drugs increase with age [7]. Without timely diagnosis, children infected with *T. cruzi* prior to implementation of vector control may miss the window of opportunity for effective chemotherapy, and the Peruvian health system may be burdened with a generation of individuals aging with Chagas disease as has occurred in other countries [8]. Due to the significant labor and cost involved in diagnosis of *T. cruzi* infection, targeted screening strategies are needed to integrate diagnosis and treatment of children into Chagas Disease control programs in South America.

Vector-borne transmission of *T. cruzi*, typically confined to rural communities, has become an urban problem in Arequipa, Peru, a city of 850,000 people. Urban transmission cycles are also established elsewhere in the region (Region of Health, Arequipa, unpublished data and [9]), but little is known about Chagas disease transmission in or around cities. The Arequipa Regional Office of the Ministry of Health began a vector control campaign in the greater metropolitan area of Arequipa in 2002, and efforts continue. We accompanied the vector control campaign to one community on the outskirts of Arequipa and collected entomological and census information as insecticide was applied to each household. We then performed a cross-sectional serological survey for *T. cruzi* infection among the children of this community.

The aim of the study was to develop targeted screening strategies to detect *T. cruzi* infection in children from data collected during a vector control campaign. Drawing on methods from ecology, traditional epidemiology and Bayesian statistics we first describe the spatial patterns of *T. cruzi* transmission in a community and evaluate risk factors for infection in children. We then show how spatial and risk factor information can be used to identify high-risk children for targeted screening and evaluate alternative targeted screening strategies.

## Methods

### Study Area and Population

Arequipa is located at an elevation of 2300 meters in southern Peru. Arequipa's climate is arid most of the year, though there is rainfall between the months of January and March. Santa Maria de Guadalupe and Alto Guadalupe (hereafter referred to together as Guadalupe) are two of hundreds of communities located on hillsides on the outskirts of Arequipa (16.44°S, 71.59°W) and have been described previously [10]. Approximately 2550 people live in 397 houses of Guadalupe over an area of 14.1 hectares (2800 households/km^2^). Three hundred and seventy-four of the 397 households were sprayed with deltamethrin powder (Bayer K-othrina, Lima, Peru) suspended in water at an intended rate of 25 mg/m^2^ by the Arequipa Regional Office of the Ministry of Health in November and December of 2004. Twenty-three households either were closed or refused insecticide treatment. At the time of insecticide application, 194 (52.0%) households were found to be infested with *Triatoma infestans*, and 72 (19.3%) were infested with triatomines carrying *T. cruzi* [10]. Guadalupe was sprayed again in April of 2005.

### Entomologic Survey

During the course of the first insecticide application to households in Guadalupe, 2 trained triatomine collectors systematically searched each room of the human dwelling, each animal enclosure, and the remaining peridomestic area for a total of 1 person-hour. Triatomines captured from each site of collection were stored separately on ice packs and taken to the University of San Agustin where they were counted by site, stage, and sex (for adults). A sample of 10 live and moribund adult and 5^th^ instar triatomines from each site of collection were examined microscopically for *T. cruzi* infection following procedures outlined in Gürtler et al. [11]. In order to evaluate household risk factors for *T. cruzi* infection in children, all sites of collection were classified as either domestic or peridomestic. Domestic sites include all sleeping, living, cooking and storage rooms of the human dwellings. Peridomestic sites include animal enclosures and all other structures in the enclosed yards surrounding the human dwellings. Household position was determined with a handheld global positioning system unit with an accuracy of 10 m (Garmin Corporation, Olathe, KS, USA). The entomologic collectors also gathered information on household materials and animal husbandry practices through a structured questionnaire. A census of the human population was performed separately by research nurses. Complete survey and laboratory sampling techniques are described in Levy et. al. [10].

### Serologic Testing

Serologic testing was carried out between August and October of 2005. All children < = 18 years old were invited to participate in the study. Trained study staff explained the study to children and their parents or guardians in schools and over the course of several meetings in the community of Guadalupe. Participants 18 years of age provided informed consent. The parents or legal guardian of all participants under 18 years of age provided informed consent and each participant 7 years or older provided informed assent. The consent form was read aloud to all illiterate parents and participants, and in these cases consent or assent was indicated with the person's fingerprint rather than a signature.

After informed consent, 5 ml of venous blood was drawn by a trained research nurse from children over 5 years old; 3 ml was drawn from younger children. Blood was kept on ice and separated on the day of collection by centrifugation. Aliquots of sera were stored at −20°C until testing. Sera were tested by commercial ELISA (Chagatek, Biomerieux, formerly produced by Organon Teknika). All positive sera, and 10% of negative sera, were tested by immunofluorescence assay (IFA) at the Centers for Disease Control and Prevention. A specimen was considered positive by ELISA if absorbance was at least 0.100 greater than the average absorbance of three negative controls, following the manufacturer's indications. IFA titres of 1∶32 or higher were considered positive (F. Steurer, Division of Parasitic Diseases, CDC, Atlanta, GA). Children whose blood was positive by both ELISA and IFA were considered seropositive [3]; three children with discordant results were excluded from this analysis. All seropositive children were enrolled in Peru's integral health system (SIS-sistema integral de salud), and offered free, directly-observed, treatment by the Arequipa Regional Office of the Ministry of Health. The study protocol was approved by the Institutional Review Boards of A. B. PRISMA, Instituto Nacional de Salud, Peru (National Institute of Health, Peru), Johns Hopkins University Bloomberg School of Public Health, and the Centers for Disease Control and Prevention.

### Data Analysis

#### Spatial Analysis

To describe the spatial distribution of vectors, parasite-infected vectors, and confirmed seropositive children we calculated the spatially smoothed density of households containing each across the study area. We used a kernel smoothing function with a Gaussian kernel of a bandwidth of 25 meters [12]. For comparison, we also smoothed the density of households without vectors, parasites and seropositive children, and calculated the ratio of the two sets of smoothed densities across the study area. We used Ripley's spatial K function to test for spatial clustering of households with seropositive children [13]. Conceptually, the K function measures the expected number of observations (in our case households) within a set distance of any given observation. We calculated the difference between the K function summarizing the degree of clustering of households with confirmed seropositive children and the K function summarizing the degree of clustering of households with seronegative children. A difference in K functions of greater than zero suggests spatial clustering [14]. We repeated the analysis at 30 spatial scales from 10 to 300 meters. For each spatial scale we constructed 99% tolerance limits around the observed difference in K functions through simulation [15]. Using the same methods we also compared the K functions of households with vectors compared to households without vectors, and households with *T.cruzi*-infected vectors to those with uninfected vectors.

### Univariate Analysis

After examining the spatial patterns of vector infestation, parasite-infected vectors and seropositive children, we evaluated the association between the presence of a seropositive child and local covariates measured in each household. All data on local covariates were collected during the insecticide spray campaign. We divided these covariates into groups based on the amount of effort needed to collect each type of data. Census data, such as age and the presence of domestic animals, required the administration of a questionnaire. Routine spray data were those data collected by the Ministry of Health during spray campaigns, such as the presence or absence of vectors in the domestic and in the peridomestic area. Timed vector search data required a systematic timed entomologic search, and consisted of estimates of vector densities in the domestic and peridomestic areas. Microscopic examination data comprised the presence of *T. cruzi-*infected vectors among a sample of insects captured from the domestic or peridomestic areas.

In univariate analyses, associations between confirmed seropositivity and binary covariates were evaluated with the χ^2^ test. For continuous covariates a non-parameteric receiver operating characteristic (ROC) curve was plotted and the area under the curve (AUC) estimated by the trapezoid rule of integration. Larger areas (greater than 50%) indicate positive association between the covariate and confirmed diagnosis; smaller areas (less than 50%) indicate negative association. The area under the non-parametric ROC curve is equivalent to the Wilcoxon rank sum statistic (also known as the Mann-Whitney U statistic), and p-values for associations between continuous covariates and confirmed seropositivity were estimated by the Wilcoxon rank sum test [16]. All covariates with p value <0.05 in univariate analysis were considered in multivariate analyses.

### Multivariate Bayesian Hierarchical Modeling

We used Bayesian hierarchical mixed modeling techniques to estimate the effects of multiple covariates on the probability of confirmed seropositivity controlling for spatial autocorrelation of the outcome variable. The mixed model conditions inferences and predictions on an unknown underlying risk of each child (a random effect) [17]. We fit a spatial conditional autoregressive logistic model in which the underlying risk of children in each household was assumed to be a function of the infection status of children in neighboring households [15]. We considered two households neighbors if they were within 42 meters of each other because *T. infestans* nymphs are known to crawl at least 42 meters [18]. One child's home had no neighbors and was left out of the analysis.

Bayesian hierarchical models have been used for many years in epidemiology, and especially spatial epidemiology (reviewed in Boyd 2005 [17]). Inference in Bayesian hierarchical models is based on the joint posterior distribution, or the posterior, of model parameters. Bayes' theorem states that the posterior distribution is proportional to the product of a conditionally independent likelihood and the prior distribution of the parameters. Prior distributions, or priors, describe the conceivable values of a parameter before the collection of the data. We specified non-informative priors for model parameters so as to minimize the importance of our *a priori* assumptions. Priors for all fixed effect parameters were normally distributed with a mean of 0 and a variance of 10^6^. We specified a Gaussian distribution for the random effects, and set a uniform prior distribution for the inverse of the standard deviation of the Gaussian distribution. Overly wide priors for the inverse standard deviation of the random effects led to numerical overflow errors; a uniform prior with a range from 0 to 15 was broad enough to avoid truncating the posterior estimates and narrow enough to avoid overflow errors. Models were fit in WinBugs 14.1; code is available upon request.

### Evaluation of Targeted Screening Strategies

We used the fit Bayesian models to rank the children in the community based on their age and local covariates, but without including any information about the spatial component of risk, which is unknown prior to testing. Using the ranking from the fit models we plotted non-parametric ROC curves and calculated the area under these curves. We then considered two-step case detection strategies that reincorporated spatial information. In two-step strategies the fit multivariate models are used to rank the risk of seropositivity in children based solely on their age and local covariates collected from their households during the spray campaign. In the first step of screening a proportion of the highest-risk children are tested. The results of the preliminary screening are used to identify children living within a given distance of seropositive children, and in the second step of screening these children are tested (ring screening). We considered ring screening radii of 10, 20, 30, 40, 50, 60 and 70 meters. For each radius we plotted an ROC curve for the two-step screening strategy and calculated the area under the curve. We calculated the sensitivity (number of cases detected/total number of cases) and specificity (number of non-cases detected/total number of non-cases) for all potential cutoff points of the ROC curves. We report the percent of non-cases (1-specificity) that must be tested to identify >80% of cases for each fit model. ROC analyses were programmed in the R statistical environment (www.r-project.org), code is available from the authors upon request.

## Results

Specimens were tested for a total of 433 children. Of these, 26 (6.0% [95% CI 3.8–8.4]) were positive for antibodies to *T. cruzi* by ELISA, and 23 (5.3% [95% CI 3.4–7.9]) were confirmed positive by IFA. No ELISA-negative specimens were positive by IFA. ELISA-positive specimens from 3 children were IFA negative; these children were excluded from subsequent analysis. Thirty-two children either lived in households that refused spraying or could not be matched to households based on address information provided by their schools. The total sample size for risk factor analyses was 398 children, of whom 23 (5.8%) were confirmed seropositive for *T. cruzi* infection.

### Univariate Associations

Of census data variables, age and the presence of animals, almost exclusively dogs and cats, sleeping in the domestic area of the household were significantly associated with confirmed *T. cruzi* seropositivity (Table 1). Both the presence of triatomines in the domestic and peridomestic areas were weakly associated with child seropositivity. Vector densities in the domestic and the peridomestic areas were associated with seropositivity, but the association was stronger for domestic vector density. The presence of *T. cruzi-*infected vectors in domestic sites of collection and in the peridomestic area were both significantly associated with seropositivity. Neither the presence of any specific animal species in the peridomestic area, nor the presence of any specific building material in the domestic area were significantly associated with child seropositivity (data not shown).

**Table 1:** Univariate risk factors for *T. cruzi* seropositivity in children aged 18 years and younger in the community of Guadalupe, Arequipa, Peru, 2005

Data Source and Covariates	Mean (range) or number of children (%) (N = 398)	Odds ratio or Area under ROC curve*	p-value*
**Group A. Census data**			
Age	10.9 (1–18)	AUC = 64.4% [54.3–74.5]	0.020
Presence of animals sleeping inside house	80 (20.1%)	OR = 2.75 [1.01–7.12]	0.019
**Group B. Routine spray data**			
Domestic infestation	216 (54.3%)	OR = 2.51 [0.02–7.92]	0.051
Peri-domestic infestation	125 (31.4%)	OR = 2.10 [0.81–5.36]	0.081
**Group C. Timed vector search data**			
Estimated domestic vector density	7.1 (0–200)	AUC = 63.2% [51.4–75.0]	0.025
Estimated peri-domestic vector density	9.7 (0–616)	AUC = 58.6% [46.90–70.3]	0.092
**Group D. Microscopic examination data**			
Domestic *T. cruzi*-infected insects	61 (15.3%)	OR = 2.60 [0.86–7.05]	0.038
Peri-domestic *T. cruzi*-infected insects	42 (10.6%)	OR = 4.25 [1.37–11.79]	0.001

*: The area under the ROC curve is presented as a percent of the total possible area to avoid confusion with odds ratios; a value of 50% is expected by chance, higher values indicate positive association with confirmed diagnosis, lower values indicate negative association. The area under the curve is equivalent to the Wilcoxon rank sum statistic (also know as the Mann-Whitney U statistic), and p-values for associations between continuous variables and confirmed diagnosis are estimated by the Wilcoxon rank sum test.

### Spatial Analyses

**Figure 1:**
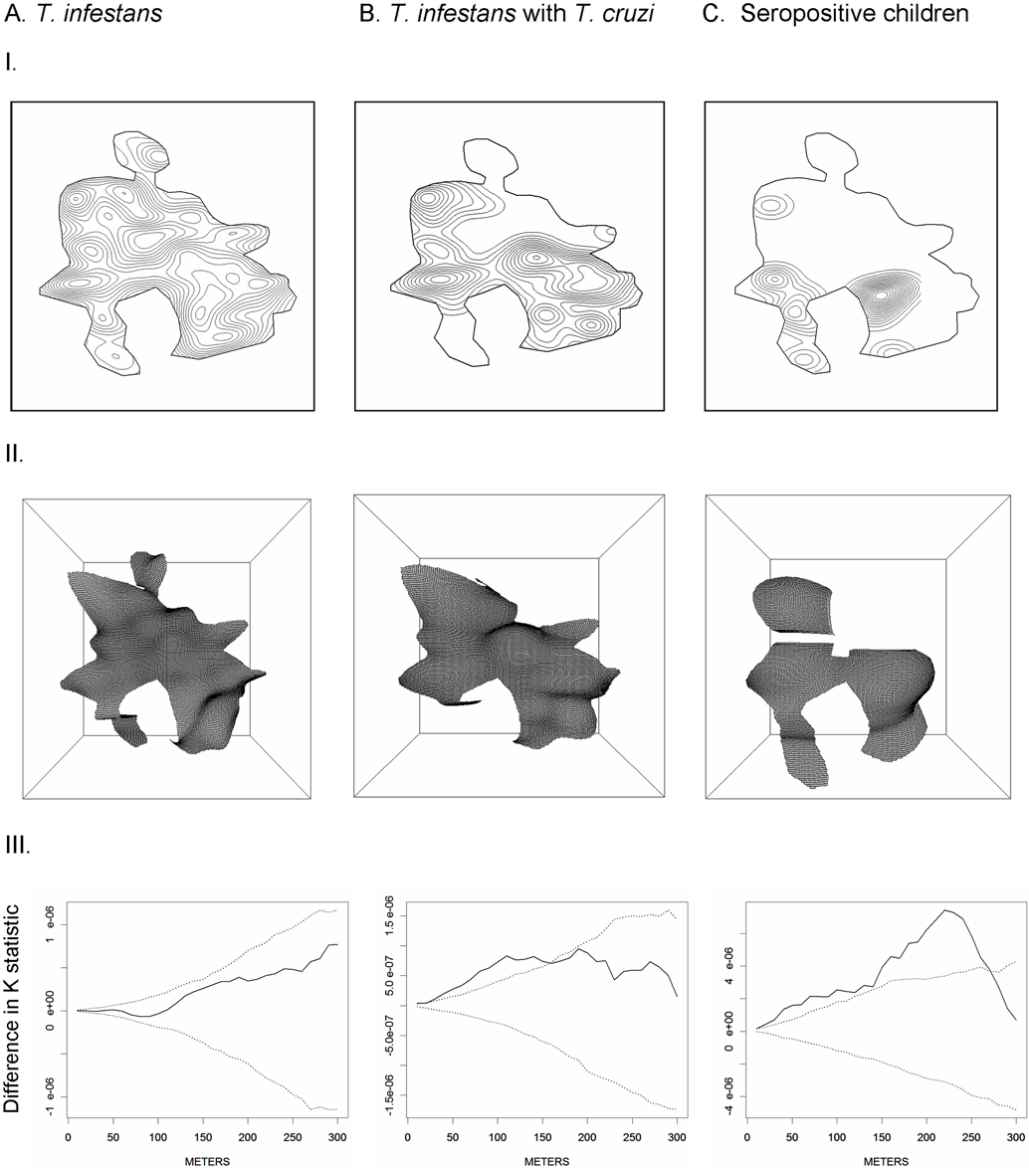
I) Contour map of smoothed density of households with A. *T. infestans*, B. *T.cruzi*-infected *T. infestans* and C. Seropositive children; II) Log relative risk surfaces of households; III) difference in Ripley's K statistic. Guadalupe, Arequipa, Peru, 2005.

The difference in Ripley's K functions for households with and without triatomines never exceeded the 99% tolerance limits, suggesting no significant spatial clustering of vector infestation (Figure 1,I-3). The difference in K functions for households with *T. cruzi*-infected triatomines and remaining households exceeded 99% tolerance limits at all but 1 spatial scale (20 m) from 10 to 140 m indicating clustering of infection in vectors within the spatial distribution of the vectors (Figure 1,II-3). The difference in K functions for households with seropositive children and those with seronegative children exceeded tolerance limits at all spatial scales from 10 to 270 m indicating significant spatial clustering of seropositivity among children tested in the community (Figure 1,III-3).

### Multivariate Bayesian Hierarchical Models

After controlling for spatial effects, the risk of *T. cruzi* infection increased 20% with each year of age (Table 2, model A). The presence of animals sleeping inside the house did not significantly increase risk after controlling for age and spatial effects. The presence of triatomines within the household increased the risk of infection nearly two-fold after controlling for age and spatial effects, but the increase was not statistically significant (Table 2, model B). The estimated domestic vector density was significantly associated with the risk of seropositivity. A child's risk of infection increased by 4% for each bug captured in the domestic area after controlling for age and spatial effects (Table 2, model C). Domestic vector densities were very heterogeneous between households. Children who lived in households in which we detected a *T. cruzi*-infected triatomine were not significantly more likely to be seropositive than those in households without *T. cruzi-*infected vectors after controlling for age, nor after controlling for age and vector density inside the house (Table 2, model D).

**Table 2:** Fit multivariate models predicting *T. cruzi* infection in children 18 years and younger in the community of Guadalupe, Arequipa, Peru, 2005

Data Source	Model	Formula	Estimated Odds Ratio [2.5%, 97.5% quantiles]
Census alone	A	α+β_age_*X_a_+φ_j_	e^βage^ = 1.20 [1.04–1.43]
		α+β_age_*X_a_+β_an_*X_an_+φ_j_	e^βage^ = 1.21 [1.04–1.45]
			e^βan^ = 2.41 [0.25–17.92]
Census & routine spray	B	α+β_age_*X_a_+β_vp_*X_vp_+φ_i_	e^βage^ = 1.21 [1.03–1.45]
			e^βvp^ = 8.40 [0.93–185.68]
Census & timed vector search	C	α+β_age_*X_a_+β_vd_*X_vd_+φ_j_	e^βage^ = 1.22 [1.04–1.47]
			e^βvd^ = 1.04 [1.01–1.09]
Census, timed vector search & microscopic observation	D	α+β_1_*X_a_+β_tc*_ X_tc_+φ_i_	e^βage^ = 1.21 [1.04–1.44]
			e^βtc^ = 2.91 [0.32–35.84]
		α+β_age_*X_a_+β_2_*X_vd_+β_tc*_ X_tc_+φ_j_	e^βage^ = 1.22 [1.04–1.46]
			e^βvd^ = 1.04 [1.01–1.09]
			e^βtc^ = 2.01 [0.17–24.75]

β's are coefficients of the fit models, X's are observations for each child. Subscripts describe the variables age = age of child, an = animal sleeping inside the domestic area, vp = Domestic vectors present, vd = Domestic vector density, tc = Domestic *T. cruzi*-infected vector present. α is the intercept of the model, and φ_j _is the spatial random effect assigned to each household (denoted by the subscript j).

### ROC Analysis of Alternative Targeted Screening Strategies

**Figure 2:**
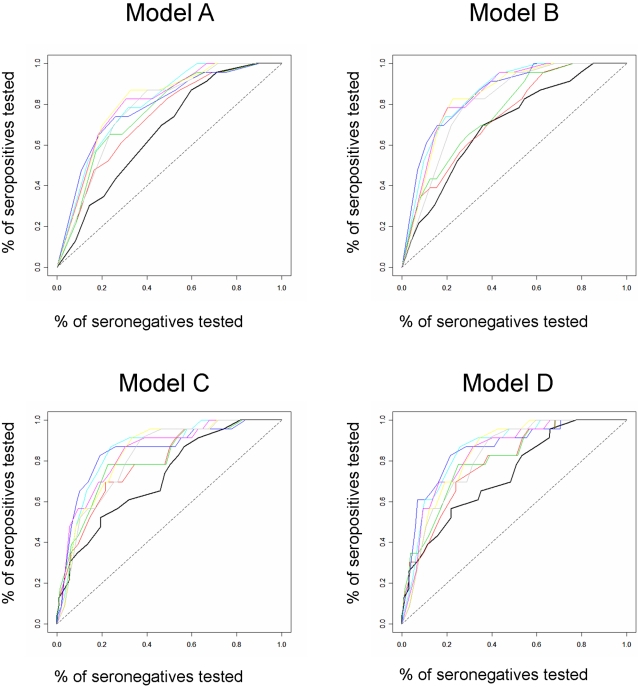
Receiver-operator curves from multivariate models predicting *T. cruzi* infection in children aged 18 years and younger in the community of Guadalupe, Arequipa, Peru, 2005. Model A includes information on age only, Model B includes data on age and vector presence, Model C includes age and domestic vector density, and Model D includes information on age, domestic vector density, and the presence of domestic *T. cruzi*-infected vectors (see Table 2). For each graph non-spatial ROC curves are in black. ROC curves for two-stage testing algorithms are in color (red = 10 meter radius, green = 20 m, blue = 30 m, teal = 40 m, magenta = 50 m, yellow = 60 m, grey = 70 m). Seropostives are positive by two tests (ELISA and IFA), seronegatives are negative by ELISA.

The area under the curve for the predictive model based on age alone was 0.64 (Table 3, model A). This area increased with ring testing, and the maximum area was 0.81 when children living within 40 or 60 meters of identified cases were also tested (Table 3, model A). Testing 28% of seronegative children would be necessary to identify >80% of infected children (Figure 2, model A). The area under the curve for the model with age and the presence or absence of vectors within the household (routine spray data) was 0.68, and also increased with ring testing to a maximum of 0.85 when the testing radius was 10 or 20 meters (Table 3, model B); >80% of infected children could be identified while testing 22% of seronegative children (Figure 2, model B). Using the timed vector search data, the model with age and household vector density had an AUC of 71% with no ring testing, and a maximum AUC of 85% when children living within 20 meters of a case were tested (Table 3, model C). Only 19% of seronegative children would need to be tested to identify >80% of infected children (Figure 2, model C). The model which included age, vector density, and the presence of *T. cruzi*-infected *T. infestans* in the household had an AUC of 72% with no ring testing and an AUC of 85% with ring testing over a radius of 10 or 20 meters (Table 3, model D); >80% of infected children could be identified by testing 22% of seronegative children (Figure 2, model D).

**Table 3:** Area under the curve for multivariate models predicting *T. cruzi* infection in children aged 18 years and younger in the community of Guadalupe, Arequipa, Peru, 2005

Covariates	Model	Area Under the ROC Curve								
		No Ring Case Detection	Radius of Ring Case Detection (Meters)							
			0*	10	20	30	40	50	60	70
Age	A	0.64	0.75	0.78	0.80	0.80	0.81	0.78	0.81	0.79
Age & domestic vector presence	B	0.68	0.75	0.85	0.85	0.84	0.84	0.81	0.81	0.80
Age & domestic vector density	C	0.71	0.80	0.84	0.85	0.84	0.83	0.82	0.81	0.79
Age, domestic vector density & domestic *T. cruzi*-infected vector presence	D	0.72	0.80	0.85	0.85	0.83	0.83	0.82	0.80	0.78

*: Ring case detection with a radius of 0 involves testing children living in the same house as identified case.

One of the two-step screening strategies with an area under the ROC curve of 85% would begin by ranking children based on their age and the number of vectors captured within their houses. In the first round of screening 15% of the highest-ranked children would be tested, and the households of all seropositive children identified. In the second round of screening all children living within 20 meters of households with seropositive children would be tested. In total 23% of the population would be screened and 19/23 (83%) of seropositive children diagnosed.

## Discussion

Chagas disease transmission cycles have become established in communities on the outskirts of the city of Arequipa, Peru. A vector control campaign is currently disrupting transmission of *T. cruzi*, but we found 5.3% of children in Guadalupe had already been infected by the time their households received insecticide application. Many thousands of children live in similar communities in Arequipa and likely represent a significant proportion of the Chagas disease burden in Peru.

We observed spatial evidence that transmission of *T. cruzi* was in an epidemic phase in Guadalupe at the time household insecticide application was initiated. As noted previously [10], although households infested with vectors were distributed across the community, those with vectors carrying *T. cruzi* were significantly clustered. Such a pattern is consistent with recent introduction of *T. cruzi* into an established vector population, followed by vector-borne dissemination of the parasite to susceptible hosts in nearby houses. Here we show further that households with seropositive children were also significantly clustered around each other. Interestingly, clusters of households with infected children were well within the looser clusters of households harboring *T. cruzi*-infected triatomines. The pattern of infected children living in households at the heart of the cluster of households with infected vectors is further evidence of epidemic spread of *T. cruzi* in the community. If the parasite is actively spreading from one or many points of introduction in the community then we would expect exposure time of children living nearer to the site or sites of introduction to be much greater than that of children living at the periphery of the parasite's distribution. This spatial inequality of exposure would lead to the observed tight clustering of infection in children.

Traditional epidemiologic methods are not well suited for epidemics when infection is clustered in space [17]. We therefore relied on Bayesian mixed models to estimate associations between risk factors and *T. cruzi* infection status, controlling for the spatial aggregation of seropositive children. We found domestic vector density to be significantly associated with infection among children living in the house after controlling for age and spatial effects. The risk of infection increased by 4% per bug found within a child's house, such an increase in risk is very important given the heterogeneity in domestic vector densities across households. We had only cross-sectional entomologic data from 2004 with which to identify associations with infections which may have occurred years earlier. Although it is unlikely that vector density is constant over time, our finding may be significant because houses with a high density of bugs in 2004 likely also had a high density of bugs in previous years. Controlling for spatial effects and household vector density, a child's risk of infection increased by 22% for each year of age; this estimate was nearly constant across all multivariate models considered.

Although no published study has described risk factors for *T. cruzi* infection in an urban epidemic situation controlling for spatial autocorrelation, our results are qualitatively similar to findings from other foci of Chagas disease. In two papers, Gürtler and collaborators described risk factors for infection in children under 16 years of age in three rural towns in northwestern Argentina where *T. infestans* and Chagas disease were endemic. The authors also found the age of children and the density of insect vectors in their houses to be significantly associated with infection [19],[20]. Similar relationships between *T. infestans* density and risk of infection have been noted by Catala [21],[22]. As in the study by Gürtler [20], we found infected children living in households in which we caught few or no vectors. Gürtler suggests various possible explanations for infection in the apparent absence of vectors, including lack of sensitivity of timed entomologic collections and infection of children while staying in other households [20]. Unreported vector control measures taken by homeowners may also be an important explanation. In the urban environment where houses are contiguous, infection by vectors entering the household from neighboring households may also pose a risk to children in houses not colonized by the vector. In Guadalupe, as in the study site described by Gürtler, there does not seem to be a threshold vector density below which transmission does not occur.

We were not able to evaluate the sero-status of participant's mothers to consider the potential that children were infected congenitally [23]. In peri-urban Arequipa, where the prevalence of *T. cruzi* infection among women of childbearing age is low [24], the risk of congenital infection is likely negligible relative to the risk of vector borne transmission. In areas with higher rates of congenital transmission infection of children by this route may decrease the sensitivity of targeted screening strategies based on spatial and household risk factors alone. A second potential cause of error for the screening strategies is prior infection of immigrant children. We were not able to gather detailed migration histories through surveys conducted during the spray campaign. Most of the children in Guadalupe were born there, and the strong spatial pattern of infection suggests that cases in children are autochthonous. In other areas, where more cases are imported, targeted screening strategies would need to rely more on migration history data.

Knowledge of local risk factors [19],[25],[26] can be used to enhance the efficiency of serologic screening by identifying groups of persons at greatest risk of infection. Previous authors have suggested identifying high-risk children by diagnosing reservoir animals in their households[19], or calculating their *Trypanosoma cruzi* transmission risk index, which includes blood meal analysis of triatomines [21]. While very important to understanding the cycle of domestic *T. cruzi* transmission, these methods are too costly for routine use in low-resource settings. We show here how entomologic data collected easily during a vector control campaign could be used to identify children at increased risk of infection. We also show that a two-step screening strategy would be much more effective in detecting seropositive children than a screening strategy based on entomological data alone. Mott et al. showed household aggregation of seropositive children in Brazil [27], suggesting that in a targeted screening campaign all children living in a house with a seropositive child should be diagnosed. In Guadalupe aggregation of seropositivity extends beyond household boundaries and testing of children living in surrounding households is needed.

Economic analysis is needed to optimize targeted screening strategies for Arequipa given the real costs of gathering data and serologic testing. Measuring vector density by the person-hour method requires that an additional person accompany each spray worker at the time of application of insecticide. Alternative methods for estimating vector density, such as leaving collection bags with homeowners following spraying [28], or timed collection by the spray worker alone, may work nearly as well at a much lower cost. Data from microscopic examination of triatomines for *T. cruzi* infection did not result in an improvement in identification of seropositive children in Guadalupe, possibly due to our ability to routinely examine only a small proportion of captured vectors. Targeted screening might be significantly simplified with the utilization of rapid tests for *T. cruzi* infection. If test results are available immediately ring screening around cases could also begin immediately, decreasing the labor involved in the two-step screening process. Economic analysis should also take into account the increase in the positive predictive value of diagnostic tests that occurs when testing is limited to a small higher-risk population.

We are limited in terms of our ability to extrapolate the findings of our study to other areas. If, as we suggest, transmission is epidemic in peri-urban Arequipa, the results of our predictive models might be sensitive to the precise timing of insecticide application. Had Guadalupe been sprayed one year later not only might the prevalence of infection have been higher, but the associations between covariates and infection might have been quantitatively different. In an analogous analysis, Struchiner et al. demonstrate how estimates of the effect of a vaccine against malaria would change over the course of a malaria epidemic [29]. The authors show that the expected effect of a vaccine decreases over the course of an epidemic, and the same may be true for local risk factors for *T. cruzi* infection. More empirical data collection and mathematical modeling are necessary to elucidate the associations between spatial, temporal and entomologic variables and seropositivity as the parasite spreads through a peri-urban community. Until we have a better understanding of the associations between risk factors and infection at different stages of epidemic transmission we suggest using adaptive sampling methodology [30] to simultaneously implement and evaluate screening strategies.

In conclusion, our results suggest that peri-urban communities in Arequipa may be in the midst of an epidemic of vector-borne *T. cruzi* transmission. Like in the early HIV epidemic, climbing prevalence of *T. cruzi* infection has gone unnoticed due to the subclinical nature of most recent *T. cruzi* infections. Ongoing vector control campaigns, in addition to preventing further parasite transmission, facilitate the collection of data essential to identifying children at high risk of *T. cruzi* infection. Concentrating diagnostic resources on these high risk children will ensure that the greatest number of infected children receive treatment before their window of opportunity for effective chemotherapy closes. Targeted screening strategies could make integration of diagnosis and treatment of children into Chagas disease control programs feasible in lower-resource settings.

## Supporting Information

Alternative Language Abstract S1 Translation of abstract into Spanish
(0.02 MB DOC)
