# Supplementary material for: Targeted Screening Strategies to Detect Trypanosoma cruzi Infection in Children
Source: PLoS Negl Trop Dis. 2007 Dec 26;1(3):e103. doi: 10.1371/journal.pntd.0000103 (PMC2154390; doi:10.1371/journal.pntd.0000103)
Supplement: Alternative Language Abstract S1 — Translation of abstract into Spanish (0.02 MB DOC) [file pntd.0000103.s001.doc]

**RESUMEN (Abstract translated by Dr. Vivian Kawai)**

**Antecedentes**

En Latinoamérica hay millones de personas infectadas con *Trypanosoma cruzi*, el agente responsable de la enfermedad de Chagas. El tratamiento anti-trypanosomico puede curar individuos infectados, pero la eficacia del tratamiento es mayor durante la infección temprana. Las campañas de control vectorial quiebran la transmisión del *T. cruzi*, pero sin diagnostico oportuno, los niños que se infectan antes de la campaña usualmente pierden la oportunidad de recibir tratamiento efectivo.

**Métodos y hallazgos**

Realizamos un tamizaje serológico en niños de 2-18 años que viven en una comunidad peri urbana de Arequipa, Perú y enlazamos estos resultados con datos entomológicos y espaciales y datos obtenidos en un censo realizado durante el control de la campaña vectorial. 23 de 433 (5.3% [95% CI 3.4-7.9]) de los niños resultaron seropositivos para la infección por *T. cruzi* utilizando dos métodos. Los análisis espaciales mostraron que las casas de los niños infectados estaban estrechamente agrupadas dentro de un grupo mayor de casas que tenían vectores infectados con el parasito. Modelos Bayesianos jerárquicos mixtos, que controlan por infecciones agrupadas, mostraron que el riesgo de un niño en convertirse seropositivo se incrementa en 20% por cada año de vida y 4% si se captura al vector dentro de la casa del niño. Los diagramas utilizando las curvas de ROC (Receiver operator characteristic) sugieren que mas del 80% de los niños infectados pueden ser identificados cuando se tamiza solo el 22% de los niños elegibles.

**Conclusiones**

Encontramos evidencia de transmisión focal-espacial del vector para *T. cruzi* en el área peri-urbana de Arequipa. Las campañas de control vectorial que actualmente se vienen realizando, además de prevenir la transmisión parasitaria, facilitan la recolección de datos esenciales para detectar niños en alto riesgo para infección por *T. cruzi.* Estrategias de tamizaje dirigidas pueden integrar los programas de diagnostico y tratamiento en niños dentro del programa de control para la enfermedad de Chagas en distritos y países de bajos recursos.
